# Changing Social Norms: the Importance of “Organized Diffusion” for Scaling Up Community Health Promotion and Women Empowerment Interventions

**DOI:** 10.1007/s11121-019-00998-3

**Published:** 2019-02-12

**Authors:** Beniamino Cislaghi, Elaine K. Denny, Mady Cissé, Penda Gueye, Binita Shrestha, Prabin Nanicha Shrestha, Gemma Ferguson, Claire Hughes, Cari Jo Clark

**Affiliations:** 1 Department of Global Health and Development, London School of Hygiene and Tropical Medicine, 15-17 Tavistock Place, London, WC1H 9SH UK; 2 University of California, Merced, Merced, CA USA; 3 Tostan International, Dakar, Senegal; 4 Equal Access International, Washington, DC USA; 5 Itad, Hove, UK; 6 Emory University, Atlanta, GA USA

**Keywords:** Social norms, Health promotion, Gender equality, Women empowerment, Low and middle-income countries, Organized diffusion, Intervention effectiveness

## Abstract

Some harmful practices are sustained by social norms—collective beliefs about what people expect from each other. Practitioners and researchers alike have been investigating the potential of social norms theory to inform the design of effective interventions addressing these practices in low- and middle-income countries. One approach commonly used to facilitate social norms change is community-based dialogs and trainings. This approach has often been criticized for not being cost-effective, as it usually includes a relatively small number of direct participants and does not allow for scaling-up strategies. In spite of some evidence (as for instance, the SASA! Program) that community dialogs can achieve social norms change, little exists in the literature about how exactly participants in community dialogs engage others in their networks to achieve change. In this paper, we look at the potential of “organized diffusion” as a cost-effective strategy to expand the positive effects of community-based interventions to participants’ networks, achieving sustainable normative shifts. We provide quantitative evidence from three case studies—Community Empowerment Program in Mali, Change Starts at Home in Nepal, and Voices for Change in Nigeria—showing that participants in community-based interventions can be effectively empowered to share their new knowledge and understandings systematically with others in their networks, eventually facilitating social norms change. Future community-based interventions intending to achieve social norms change would benefit from integrating ways to help participants engage others in their network in transformative conversations. Doing so has the potential to generate additional impact with little additional investment.

## Introduction

Social norms—the unwritten rules of acceptable behavior shared by members in a group—can contribute strongly to group members’ choices and actions. Scholars and practitioners working to improve global health and promote equitable development are currently investigating how social norms theory can inform the design of prevention interventions in low- and mid-income countries (LMIC) (Cislaghi and Heise 2017). In the last few years, non-governmental organization (NGO) practitioners have implemented programmatic strategies to influence social norms related to a wide range of gender and health-related behaviors in LMIC, such as contraceptive use (Costenbader et al. 2017); child marriage (Lee-Rife et al. 2012); female genital cutting (FGC) (Mackie and LeJeune 2009); and intimate partner violence (Shakya et al. 2017). An effective tool to change social norms are “community discussions,” where members of the same group identify local harmful practices and the norms that sustain them, eventually renegotiating both to achieve greater health, well-being, and empowerment for themselves and others in their group (Linos et al. 2013). Due to the time required, however, community discussions are often criticized by those who claim they are limited in reach. Practitioners are thus left with the dilemma of trying to engage both the greatest possible number of people in each given group of their interventions, and the largest possible number of groups in the region or country where they are working.

The aim of this paper is to examine how “organized diffusion”—the sharing of knowledge encouraged by practitioners and led by program participants—can be a fruitful strategy to increase the reach of community discussions, ultimately helping interventions achieve effective social norms change. Little empirical work has been done to examine how intervention-led diffusion processes can result in normative change, health promotion, and empowerment in LMICs. Historically, interest in diffusion in development studies and health promotion mostly focused, respectively, on the adoption of new technologies to improve rural agrarian practices and on the spread of new health-related knowledge (Greenhalgh et al. 2005). However, interest in how diffusion of information shapes culture and behavior is not new in the social sciences. Since Rogers’s (1962) foundational book (*The Diffusion of Innovations*); theorists in communication science; marketing (Robertson 1967); political science (Mintrom 1997); sociology (Katz et al. 1963); and business studies (Nonaka and Takeuchi 1995), to cite a few examples, have shown interest in understanding how new ideas gradually take hold in groups of people (for a review, see Greenhalgh et al. 2005).

We ground our analysis of how organized diffusion can contribute to social norms change on data from three case studies, respectively from three different interventions: (1) the Community Empowerment Program (CEP), implemented in Mali; 2) change starts at home (change), implemented in Nepal; and 3) Voices for Change (V4C), implemented in Nigeria. To do so, we first review key theoretical concepts. Next, for each intervention, we explain its structure, look at the methods used for data collection, and present key results. In the discussion session, we draw theoretical and programmatic implications. Concluding remarks summarize key messages emerging from our comparative analysis.

## Background

Great attention is being paid to the role that “social norms” play in influencing behaviors that shape people’s ability to protect their health and achieve their life potential. Recent attention to using social norms theory to achieve change emerged, in part, from the realization that changing harmful practices through factual information and economic inducements alone is not effective (Gelfand and Jackson 2016; Kumar et al. 2015).

Many theories of what social norms are and how they influence behavior exist (Bell and Cox 2015). Most of the literature refers to Cialdini’s theory, that identifies two types of normative beliefs: (1) one’s belief about what others in one’s group do (called *descriptive norms*) and (2) one’s belief about what others in the group approve and disapprove of (called *injunctive norms*) (Chung and Rimal 2016; Mackie et al. 2015; Miller and Prentice 2016). People comply with social norms for various reasons, including, for instance, because they are uncertain about what is the best behavior to achieve something in a given situation, they want to express membership in a group; they anticipate a social reward, or because they are forced to by those who have power over them (Bell and Cox 2015). Theorists suggest that to change a social norm it is critical to reach out to people’s “reference group,” that is, it is critical to engage the entire network of those who share the norm in question (Mackie et al. 2015; Miller and Prentice 2016; Saxena 1971).

While in high-income countries “social marketing” approaches that aim to change social norms by correcting people’s misperceptions about what others do are often chosen to achieve social norms change (Berkowitz 2010; Gidycz et al. 2011; Miller and Prentice 2016; Stock et al. 2014). In LMICs, two (sometimes intersecting) main intervention strategies are most commonly found: wide-reaching media campaigns that often incorporate social marketing strategies (Tankard and Paluck 2016) and participatory discussions between members of the same reference group (Vaitla et al. 2017). Both approaches have possible shortcomings. Community-based discussions might have limited reach due to the relatively elaborate and resource-intense nature of the intervention. While media campaigns can fail to reach the intended audience or to spark the public dialog needed for people to change their perceptions about what others in their group do and approve of due to the fairly unidirectional nature of media broadcasts.

To overcome the former challenge, some practitioners have tested a participant-led method to share information with non-participating members of their group. This method is commonly known as “organized diffusion.” Studying how the CEP facilitated change in social norms supporting FGC in West Africa, Mackie and Lejeune identified six phases in the process of diffusion of the knowledge (Mackie and LeJeune 2009). Phase one includes the discussions happening before the program, as rumors about the intervention generate curiosity. Phase two refers to the creation of the new knowledge with a selected group of participants. In phase three, participants share their knowledge with one “adopted” member in their community: usually a family member with whom they discuss what was interesting to them during project activities that day. Then, in phases four to six, information spreads out from the intervention community to new communities, eventually reaching people across the entire larger group (ethnic group, region, or country).

To our knowledge, until today, the potential of organized diffusion for normative change in LMICs was mostly theoretical. The literature on programs that facilitate normative shifts does not explicitly examine organized diffusion, a process different from but related to “community mobilization.” Community mobilization is the final piece of organized diffusion, where participants raise awareness and generate community action in theirs and other communities. Very few programs formally integrate participants’ continuous and sustained sharing of new understandings with their immediate and larger social networks in their strategy, and, as a consequence, few studies have looked at the potential of this piece of an organized diffusion process. One notable exception is the evaluation of the SASA! intervention in East Africa that achieved change in the norms supporting domestic violence through community mobilization (Abramsky et al. 2014). When Abramsky and colleagues evaluated the SASA! program, however, they did not tease out specifically the contribution of the mobilization component. Later, Starmann et al. (2018) conducted a secondary data analysis to look at how the communication materials specifically contributed to SASA!’s success, finding that radio programs and interpersonal communication contributed to the change. The importance of organized diffusion also emerged in other iterations of the program. Studying an adaptation of SASA! in Rwanda, Stern and colleagues (Stern et al. 2017) found that visibility of change helped increase organized diffusion, eventually changing the behavior of nonparticipating members of the community, even when they personally disagreed with the new behavior. Their finding that organized diffusion can first change either people’s attitudes or practices echo those from a qualitative study of the CEP (Cislaghi et al. 2016).

Despite this evidence suggesting that organized diffusion does work, we have limited evidence (especially from interventions other than SASA!) that can help elucidate to what extent organized diffusion—among other strategies—is contributing to social norms change. This paper is, to our knowledge, one of the very few that specifically examines organized diffusion across multiple programs to assess its importance. Using a comparative case method (Goodrick 2014), we examine the effectiveness of organized diffusion to achieve social norms change across three different norms change interventions in three different contexts (The CEP in Mali, Change in Nepal, and V4C in Nigeria). We provide relevant background information, explain our data collection and analysis methods, and present results on organized diffusion by case. This information is also summarized in Table 1 below, as an introductory overview to the three case studies.

**Table 1:** Overview of methods used in the three case studies

Intervention name	Study sample	Country	Intervention components	Outcome of interest	Measure of diffusion	Timing of data collection
CEP	1796 (adult women and men)	Mali	30-month curriculum on democracy, human rights, problem solving, hygiene and health, literacy, and numeracy; Community Management Committee; family and community mobilization	Family norms towards FGC	Change in normative expectations at family and community level	Baseline (2013) Midline (2015) Endline (2017)
Change	1071 (adult women)	Nepal	Radio drama; couples group work on gender norms, gender-based violence, life skills, and conflict resolution; extended family and community mobilization	Provision of support to survivors of violence	Community-level sum of discussions with others after exposure to anti-violence against women messaging	Midline/end of activities (2017)
V4C	4790 (16/25-year-old women and men)	Nigeria	12-week Safe Spaces gender curriculum; radio, advertising, social media; political advocacy	Multiple gender-related attitudes	Change in attitudes of Safe Spaces participants and peers compared to general youth population	Baseline (2014) Endline (2017)

## Case Study 1: the CEP

The NGO Tostan is a veteran of community-led social norms change in West Africa (Cislaghi 2018; Gillespie and Melching 2010; Kuenzi 2005). Their CEP, implemented since the 1990s in thousands of villages in rural West and East Africa, aims to support communities in achieving self-identified goals, which include reducing child marriage (Cislaghi et al. 2017; Jewkes et al. 2015; Michau, Horn, Bank, Dutt,, and Zimmerman 2015; Warburton 2014). Despite achieving positive results across a range outcomes including governance, education, health, environment, and economy (Cisse et al. 2018), the NGO gained considerable attention for its impact on FGC (Gillespie and Melching 2010) documented through a randomized trial (UNICEF 2008) and qualitative studies (Cislaghi 2017; Cislaghi et al. 2015).

The CEP has three components. The first is a 30-month curriculum on democracy, human rights, problem solving, hygiene and health, literacy, and numeracy. Approximately, 40 men and women take part in an informal education program that makes use of participatory pedagogical strategies (Bajaj et al. 2016; Cislaghi et al. 2017). The educational classes furnish a space where individual capacities, skills, and aspirations can grow, where group member norms can be renegotiated, and where economic constraints can be addressed, through the creation of group strategies for revenue generation. The second key component of the CEP is the Community Management Committee, a 17-person community group whose task is to implement the vision emerging from the classes in collaboration with the whole community. The third component is organized diffusion. Class participants share their learning with peers and family members, and committee members raise awareness throughout their locality. Together, participants and members of the committee also organize community mobilization activities in their villages and, eventually, in neighboring villages, motivating people living in those villages to join in the process to change local harmful norms. Intervention staff report that the engagement of local politicians and religious leaders also greatly contributed to the success of their program.

### Case Study 1 CEP: Methods

#### Sample

Tostan administered surveys in eight participating communities and four control communities at baseline (2013), midline (2015), and endline (2017) in the Kulikoro District, Mali. In total, across the three waves, 1796 respondents were surveyed. Sampling was stratified by sex, age (18–30; 30–45; 45+), and type of participation in the CEP. This last category included four possible types of participation in the CEP: (1) class participant in a CEP community, (2) adoptee in a CEP community, (3) nonparticipating person in a CEP community, and (4) nonparticipating person in a nonparticipating community. In each CEP community, sampling included 50% (*n* = 20) of the class participants and 20 adoptees, 1 per participant to understand the effectiveness of the organized diffusion strategy. The potential respondents from the CEP class were selected randomly from class rosters, stratified by age and sex as mentioned above. Adoptees were sampled following the indications given by participants in the sample of whom they had decided to adopt. Potential respondents from CEP and non-CEP communities, also stratified by age and sex as mentioned above, were selected using household listing.

#### Measures

To measure injunctive norms sustaining FGC, respondents were asked, “what would be the reaction of your family members if they knew you were going to cut your daughter,” with four possible answer choices: (1) positive reaction; (2) indifference; (3) negative reaction (obtained aggregating two possible response modalities: (i) people would disapprove of me and (ii) people would try to stop me). Respondents were also asked whether they had spoken with their family about FGC.

#### Analysis

**Fig. 1:**
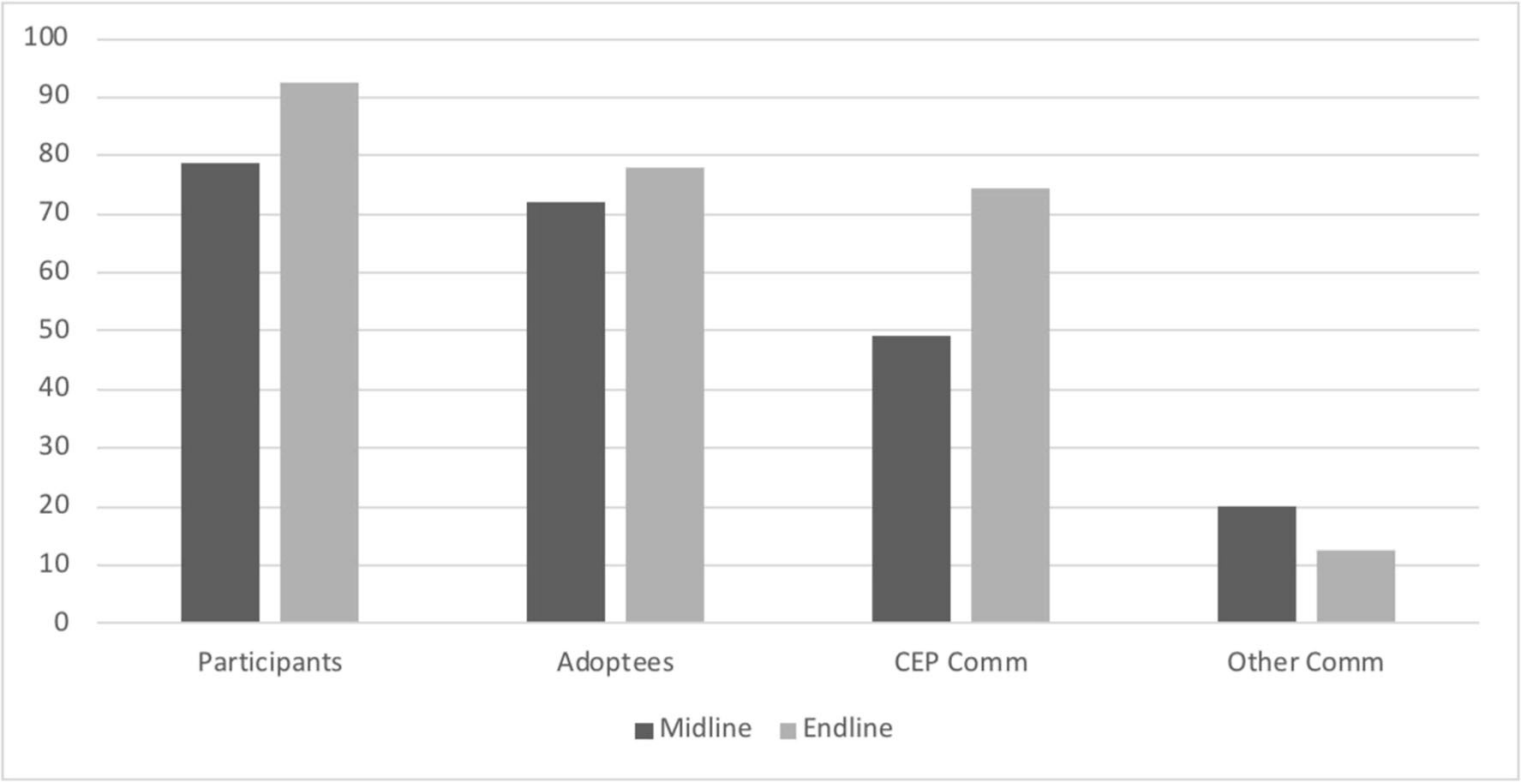
Participants anticipating family disapproval if they practiced FGC by type of participation (case study 1—CEP)

For each of these questions, we calculated response option frequencies stratified by time period (Table 2) and then further by participant types and time period (Fig. 1). Disaggregated data is only available at midline and endline, since at baseline participants had not enrolled in the program yet. The methods used as part of the evaluation of the CEP are fully detailed in Cisse et al. (2018).

**Table 2:** Injunctive norms in the family for FGC, all Sample (case study 1—CEP)

Anticipated family reaction	Baseline	Midline	Endline
Positive	84.06 (269)	35.11 (178)	25.31 (245)
Negative	14.69 (47)	60.75 (308)	62.71 (607)
Indifferent	1.25 (4)	4.14 (21)	11.98 (116)

Participants were asked what kind of reaction they anticipated from their family if they told them they were getting their daughter cut (e.g., “they would gossip about it;” “they would disapprove of me;” or “they would congratulate me”). They were then asked to categorize this reaction as positive, negative, or indifferent

### Case Study 1 CEP: Results

Table 2 presents the anticipated family reaction by time period. At baseline, 84% of respondents anticipated positive reactions in their families for cutting their daughters. At midline, this percentage had, on average, decreased to 35%.

However, when disaggregated by type of participation in the program, the data show that, respectively, injunctive norms decreased more across participants and adoptees (21 and 23% respectively) than across other members in intervention communities (43%) and members of control communities (where there is no significant change). Figure 1 below shows the percentage of respondents who anticipated negative injunctive norms from their family members.

These descriptive results suggest that, at midline, the organized diffusion had begun to shift norms within the family, as witnessed by the fact that injunctive norms in the family changed for participants and adoptees (members of the same families), less for other respondents in the intervention communities, and not at all for control communities. At endline, after participants’ efforts to share knowledge at large in the intervention communities, we observe further decrease in family approval for FGC (5% participants, 15% adopted members, and 8% other members in intervention communities), with no change in control communities.

These descriptive results also offer an indication that these changes in injunctive norms within the family were associated with participants having talked to other members in their families. At midline, 69% of participants reported having actively talked to their family about FGC, while 35% of adoptees did so, which speaks to the fact that at midline adoptees were mostly recipients of information, rather than diffusors. This percentage decreases to 27% for other members in CEP communities and 12% in control communities. At endline, the percentages of participants and members in control communities who talked to their families about FGC remained similar; however, it increased for adoptees (up to 48%) and other members in CEP communities (up to 33%). This increase suggests that, after midline, information recipients became active diffusors too, contributing to the change in social norms described earlier.

## Case Study 2: Change

Change is a multicomponent social behavior change communication and community engagement strategy designed to prevent male-perpetrated intimate partner violence (IPV) against women in Nepal (Clark et al. 2017). The intervention was designed and implemented by Equal Access International (EAI) as part of a randomized controlled trial designed and run by Emory University. Change seeks to shift attitudes, norms and behaviors that underpin IPV perpetration in Nepal, positing that “changes in social norms can successfully promote sustainable change that protects women from IPV” (Heise 2011).

Change’s strategy has four core components: (1) a 39-week, “edutainment” radio program involving drama and discussion elements; (2) a 40-week “couples” curriculum delivered to 360 married couples via weekly facilitated Listening and Discussion Groups (LDGs); (3) wider community engagement largely through LDG organized activities; and (4) trainings for religious and community leaders (Clark et al. 2017). Informed by both the radio content and the curriculum and guided by EAI trained local facilitators, the LDGs provide a safe space for couples to critically reflect on existing harmful gender norms and renegotiate more mutually respectful relationships.

The pathway to change envisioned by the intervention follows three distinct phases: (1) a critical reflection phase; (2) a skill-building phase (where couples are exposed to and learn new life skills); and (3) a community mobilization phase, which encourages organized diffusion through community actions delivered by the LDGs. Throughout the three phases, LDG members are encouraged to share what they learn in the group meetings with family members, who are also invited to join the group sessions at three time points over the course of the 9-month intervention.

### Case Study 2 (Change): Methods

#### Sample

The study is a pair-matched, repeated cross-sectional two-armed, single-blind cluster trial (*N* = 36 Village Development Committees (VDCs), 1440 married female respondents randomly selected from the communities, 360 married female LDG members) conducted in three districts in Nepal (Chitwan, Kapilvastu, Nawalparasi). Data for the present study stem from the midline survey (12 months post baseline), and was further restricted to intervention communities (*N* = 18 VDCs; 1070 adult, female, married, reproductive age respondents) to enable assessment of diffusion and to avoid proxy tests of intervention impact in an ongoing trial.

#### Measures

The outcome variable was measured with a single item inquiring whether the respondent had provided support to a partner violence survivor in the prior 12 months (yes or no). Direct exposure to anti-violence against women messaging (anti-VAWG) was measured by five items asking if the respondent had heard a message about violence between a husband and a wife through each of five message sources (radio, television, theater, film, community leader) in the past 12 months. A dichotomous measure of direct exposure was created as a “yes” to any of the five message types or “no” if none of the five message types had been heard. For each message source, the participant was provided with prespecified options ranging from spoke to no one about the message to 14 types of family members, friends, neighbors, and other. A total diffusion score was calculated as a sum of the interactions with others across all of the message sources averaged to the community (ward) level. A measure of high versus low diffusion was calculated with high being greater than the 50th percentile (3.94 persons spoken to) of the ward-level distribution.

#### Analysis

The analysis strategy included the use of generalized estimating equations with a logit link to examine the relationship of exposure to anti-VAWG messages (direct and living in a high diffusion community) on participant report of assisting an IPV survivor in the prior 12 months, adjusting for respondent’s education level and being an LDG group member (main effects model). In a second model, an interaction term between direct message exposure and level of community diffusion was also included to examine whether the relationship between direct message exposure and survivor assistance differed by whether the person lived in a community in which diffusion was extensive (interaction model). A full description of these methods is also available in Clark et al. (2017).

### Case Study 2 (Change): Results

To understand the effectiveness of the organized diffusion component in change, we looked at diffusion of anti-violence messages and the impact of this diffusion among community members who did and did not directly hear the messaging. In the intervention communities, 67.01% (*N* = 717) respondents had heard at least one message directly. Of those hearing a message, participants were exposed to 2.03 message sources on average (SD = 1.05; range, 1–5). Across the various message types, participants were exposed to anti-VAWG messages most often from the television, followed by the radio, film, and least frequently from a community leader. Among those who heard a message directly, 76.57% (*N* = 549) spoke to someone about it, most often a neighbor, followed in frequency by a friend and then spouse. Ward-level average diffusion scores ranged from 1.57 to 11.07 persons spoken to demonstrating rather significant differences in degree of diffusion.

Among the respondents, 18.41% (*N* = 197) reported personally trying to help a married woman who had been beaten or otherwise hurt by her husband in the prior 12 months. In the main effects model (Table 3), direct exposure to anti-violence messaging was associated with providing assistance to an IPV survivor in the past 12 months (OR, 2.36; 95% CI, 1.43, 3.90). Living in a high diffusion community was not (OR, 1.20; 95% CI, 0.83, 1.72). In the interaction model, the interaction between direct message exposure and living in a high diffusion community was significant, suggesting that the relationship between diffusion and assistance to a survivor depended on whether the respondent was directly exposed to the message or not.

**Table 3:** Relationship between living in a high diffusion community and providing assistance to a partner violence survivor (*N* = 1070) (case study 2—change)

	Main effects model			Interaction model			Stratified model					
							No direct exposure to message			Direct exposure to message		
	OR	95% confidence limits		OR	95% confidence limits		OR	95% confidence limits		OR	95% confidence limits	
Direct exposure to message	2.36***	1.43	3.90	7.14***	2.93	17.40						
Live in high diffusion community	1.20	0.83	1.72	5.10***	1.97	13.18	5.24***	1.93	14.19	0.90	0.61	1.34
Direct exposure to message × live in high diffusion community				0.18***	0.06	0.50						
Educational attainment	1.09*	0.99	1.21	1.10*	0.99	1.21	0.99	0.84	1.17	1.12*	0.99	1.26
Listening and discussion group member	1.35*	0.94	1.94	1.39*	0.96	2.00	1.29	0.34	4.93	1.39*	0.96	2.02

*p < .10 **; *p* < .05; ***p < .01

To represent the findings more clearly, we present the main effects model stratified by whether the respondent directly heard a message or not. Living in a high diffusion community was associated with greater odds of assisting a survivor only among individuals who were not directly exposed to an anti-VAWG message (OR, 5.24; 95% CI, 1.93, 14.19). There was no additional benefit of residence in a high diffusion community among people who had heard a message directly (OR, 0.90; 95% CI, 0.61, 1.34). They were already more likely to support a survivor regardless of the level of organized diffusion. However, among those who were not directly exposed to an anti-VAWG message, living in a community with more extensive organized diffusion was associated with assisting a survivor.

## Case Study 3: V4C

V4C was an innovative 5-year program (2012–2017) implemented in four of Nigeria’s states (Enugu, Lagos, Kaduna, and Kano) and focused on changing young people’s attitudes and practices in three main behavioral areas: violence against girls and women, support for women’s role in household decision-making, and support for women’s political leadership. The program sought to create change at three levels: (1) among individuals, through “Safe Space” gender courses offered to young women and men online and in person in selected higher education institutions in the focal states. In these Safe Spaces, young people were encouraged to reflect on and debate gender issues and take actions to promote gender justice; (2) in wider society, through a branded communications campaign targeting young people through radio discussions and dramas, TV, social media, and billboards; and (3) within formal institutions, through the passage of legislation enhancing women’s rights and supporting women’s participation in political structures. In such a way, V4C intended to catalyze agents of change, ready and able to diffuse their new attitudes and behaviors among peer networks and create the societal space for them, and others, to adopt more equitable gender behaviors. By virtue of their post-secondary education, the students were well-positioned to influence others in their home communities (Welsh et al. 2017).

### Case Study 3 (V4C): Methods

#### Sample

V4C target population included male and female Nigerian youth aged 16–25 in four target states (Enugu, Kaduna, Kano, and Lagos). To obtain a random sample, 464 enumeration areas were randomly selected using a list from the last census in 2006. To ensure that every male and female between the ages of 16 and 25 had equal likelihood of selection, a household listing of young people in the target age range was undertaken prior to the survey enumeration. Ten to 12 respondents (half male and half female) from each enumeration area were randomly selected from the household listing. Baseline (2014) response rate was 99% (*N* = 4766). Two midline surveys were conducted in 2015 and 2016. By endline (2017), the sample had aged to represent young adults ages 19–28. The recontact rate between 2014 and 2017 was 82% (*n* = 3926). In the endline study (Denny and Hughes 2017a), we also oversampled young people with direct involvement in V4C interventions like Safe Spaces (*n* = 2147) to more accurately measure changes in gender attitudes and behaviors in this small percentage of the population (total endline *n* = 6073) and to enable us to compare the program effects on direct beneficiaries and those reached through the branded communications. A full description of question methodology, sampling, design effects, and weights is presented elsewhere (Denny and Hughes 2017a).

#### Measures

At baseline and endline, respondents were asked about gender-related attitudes, practices, and expectations, particularly in three areas thought to be influenced by gender-related social norms: physical violence against women and girls, women standing for local leadership positions, and women sharing household decisions with men (Denny and Hughes 2017b). At baseline and endline, survey respondents were asked to place themselves on a 9-point scale based on how much they influenced people around them. Since V4C’s baseline analysis suggested that behaviors such as violence against women and decision-making were also shaped by norms around speaking out against violence, sharing gender information, and challenging the status quo (Denny and Nwankwo 2015), the endline study measured self-reported changes in willingness to speak out against harmful or unequal treatment of women. Recognition of different V4C interventions was also measured on the endline questionnaire. If respondents recognized Safe Spaces specifically, they were asked if they had personally participated in them or if they knew someone who had participated. Distinct levels of V4C program exposure include Safe Spaces participants (direct, in-depth exposure), peers of Safe Spaces participants (secondary exposure), exposure to population-wide branded communications (a lighter exposure), and no exposure to any programming.

#### Analysis

To understand the effect of organized diffusion, we compared attitudinal and behavioral changes for Safe Spaces participants and their peers to changes among other young people (reached through the branded communications campaign). Models regressed baseline to endline change in the outcome variable on program exposure level (Safe Spaces, peers of Safe Spaces participants, and young people reached through the branded communications). Regressions controlled for baseline level of the outcome variable, gender, state, and age; standard errors were clustered by enumeration area.

### Case Study 3 V4C: Results

For every Safe Spaces participant, 3.8 more respondents knew a participant (in the Safe Spaces) though they did not participate themselves (range, 0.22–41.52). Direct Safe Spaces participation correlated with the most positive endline responses across the seven indicators (Table 4). Table 4 also shows that peers of Safe Spaces participants were significantly more likely to hold positive gender attitudes and report more change over time than young people with exposure through the branded communications only. Attitude and behavior change among peers of Safe Space participants was generally a half to a third as large as that for Safe Space participants themselves. The former’s attitude and behavior change was also at least 2–3 times larger and consistently more significant than changes among those reached through V4C’s branded communications only. This indicates that while in-depth programming remains most effective for participants, secondary effects among participants’ peers can also be significant when compared to the population-wide branded communications.

**Table 4:** Self-reported attitudes and behaviors for key gender issues: endline levels and change in past 2 years (case study 3—V4C)

	(1) Change: contemplating gender issues	(2) Women deserve equal opportunity and respect	(3) Support female leaders	(4) Change: support female leaders	(5) Will speak up against VAWG	(6) Change: will speak up against VAWG	(7) Others should challenge women’s limitations
Participated in Safe Spaces	0.962***	0.526***	0.489***	0.547***	0.521***	0.543***	0.492***
	(0.0284)	(0.0276)	(0.0247)	(0.0102)	(0.0239)	(0.00963)	(0.0310)
Heard of Safe Spaces via peers	0.521***	0.287***	0.193***	0.0965***	0.110*	0.0933***	0.222***
	(0.0740)	(0.0594)	(0.0715)	(0.0229)	(0.0618)	(0.0214)	(0.0705)
V4C “light-touch” exposure	0.280***	− 0.00834	0.0333	0.0323***	− 0.0484*	0.0268***	0.00832
	(0.0348)	(0.0325)	(0.0312)	(0.0100)	(0.0281)	(0.00968)	(0.0350)
Constant	0.667***	1.313***	1.284***	0.377***	1.454***	0.404***	1.361***
	(0.0741)	(0.125)	(0.141)	(0.0327)	(0.0596)	(0.0217)	(0.105)
Observations	6022	6061	6034	6073	6038	6073	6001

OLS regression models include controls for age, gender, and state. Clustering by enumeration area. Standard errors in parentheses. *p < .1; **p < .05; ***p < .01

Due to the nature of self-reported data, different groups might have different starting levels of gender awareness or might inaccurately recall how their attitudes have shifted over time. To overcome this concern, we leverage our panel data to measure how much participants’ responses to key gender questions changed from baseline to endline. Table 5 shows consistently strong positive attitude and behavior change among peers of Safe Spaces participants in the three behavioral areas of women’s political leadership, violence against women, and women’s participation in household decision-making. Results for Safe Spaces participants themselves are likely underpowered as they comprise < 1.5% of young people in the population representative survey. In each panel round, target attitudes and behaviors were measured on a 5- or 4-point scale (columns 1–4 and 5–6, respectively). We regressed the difference between participants’ endline and baseline responses on program exposure to assess how many more points along the response scale that cohort moved (2014–2017), compared to respondents with no V4C exposure.

**Table 5:** Change in key gender attitudes and behaviors, difference in reported levels at endline and baseline (case study 3—V4C)

	(1) I want to lead: change (5-point scale)	(2) Women do lead: change (5-point scale)	(3) Women should lead: change (5-point scale)	(4) Woman’s opinion matters: change (4-point scale)	(5) Woman’s opinion should matter: change (4-point scale)	(6) Appropriate to hit woman: change (5-point scale)	(7) Influence: change (9-point scale)
Participated in Safe Spaces	− 0.241	0.0357	− 0.191	0.0573	− 0.0702	− 0.154*	0.285
	(0.232)	(0.157)	(0.185)	(0.0632)	(0.116)	(0.0820)	(0.253)
Heard of Safe Spaces via peers	0.425***	0.359***	0.415***	0.111***	0.209***	− 0.299***	0.938***
	(0.143)	(0.127)	(0.0834)	(0.0424)	(0.0585)	(0.0420)	(0.160)
V4C “light-touch” exposure	− 0.124*	0.110**	0.166***	0.0812***	0.0810***	0.0349	0.283***
	(0.0664)	(0.0444)	(0.0471)	(0.0222)	(0.0251)	(0.0331)	(0.0919)
Constant	2.932***	1.569***	2.900***	1.982***	1.790***	0.390***	4.816***
	(0.210)	(0.169)	(0.175)	(0.0884)	(0.0961)	(0.105)	(0.242)
Observations	3894	3707	3830	3335	3593	3639	3926

OLS regression models include controls for age, gender, state, and 2014 level of dependent variable. Clustering by enumeration area. Standard errors in parentheses. *p < .1; **p < .05; ***p < .01

The positive change among these young people with secondary exposure to in-depth programming is not as large as direct contact with the program. However, across a range of gender norms measures, both self-reported and objective change is larger for peers of Safe Spaces participants compared to young people with exposure to the branded communications only or no exposure at all. It is likely that because gender awareness and leadership skills are both diffusing through social networks, peers were simultaneously gaining insights into their community’s descriptive and injunctive norms and how they may be changing.

Diffusion of Safe Spaces awareness also corresponded with an increase in how much influence young people say they have on the people around them (Table 5, Column 7), a key component to catalyzing community change. By program completion, peers of Safe Spaces participants showed an increase in perceived influence nearly a full point larger (on the 9-point scale) than young adults with no program exposure—and 0.66 points larger than young adults with exposure through the branded communications only. This increase suggests that through diffusion, peers of Safe Space participants are growing in influence and knowledge in ways that could empower them to further spread gender attitudes and behaviors.

## Discussion

Results from the three case studies show the potential for organized diffusion. The three sets of results suggest that facilitating a process through which participants share their knowledge with others can help achieve change in existing social norms, ultimately contributing to change in their practices. Existing theory can assist us to interpret study findings.

Neo-diffusionism (Kashima 2009, 2014) suggests that through communication, ideas are passed from one cultural agent to others. For this communication to affect the listener’s opinions, the speaker needs to tailor an appropriate message. Through the conversation, the speaker and the listener agree on the understanding of what they talked about, strengthening both the listener’s and the speaker’s beliefs relative to their conversation. Across the three case studies, our findings corroborate Kashima’s central tenet, suggesting that intracultural processes of diffusion can be facilitated when the listener and the speaker know each other well (for experimental evidence, see Lau et al. 2001). In the CEP case study, we showed that diffusion first happened in the family, where the speaker’s capacity to assess the listener’s knowledge and to anticipate their reactions is, on average, likely higher than with others in the rest of the community. In the Change study, the most prominent persons with whom the participant discussed anti-VAWG messaging was a neighbor, family member or husband. In the V4C study, the peers of directly exposed participants exhibited significant change, just on a lesser scale than those directly exposed. Neo-diffusionism also purports the more frequent the communication, the more the new information will spread across the social network, evolving into a new reality. As the new understandings become meaningful, not only to individuals but also to communities as a whole, they embody more than new emerging beliefs: they become standard acceptable ways of making sense of the world shared within one’s social group, eventually changing how members of that group think and act. In our data, we found that organized diffusion increased the positive changes in behaviors that were sustained by harmful social norms, suggesting that the new understanding and knowledge were indeed becoming part of a new shared social narrative of acceptable actions.

In addition to the theoretical literature on diffusion of knowledge within complex cultural systems, the literature on social movements can be of further assistance in interpreting the significance of the findings emerging from our case studies, as it incorporates greater awareness of the resistances faced by those who attempt to change an unequitable status quo. Christiansen (2009) argued that social movements have four key stages in their life cycle: (1) emergence (widespread discontent); (2) coalescence (population collective aware of widespread discontent); (3) bureaucratization (the formalization of the movement into an organization); and (4) decline (the end of the movement either because it succeeded or was repressed). Our findings shed greater light on how social movement move from phase 1 to phase 2. We found that a core group of motivated activists is needed to and effective at increasing individual and collective awareness of a widespread discontent with the current status quo. Then, as those motivated activists reach out to others in their community, their new understandings of how things could be different can facilitate coalescence of intents. At this point, a general sense of individual unease becomes more concrete: it gets discussed in conversations that generate new visions, strengthening people’s collective intentions to address what is causing it. Together, diffusion theory and the four stages of social movement model offer a theoretical explanation to why we found very similar patterns across three cases studies, with important implications for practice.

Collectively, the key message emerging from our findings is that integrating organized diffusion strategies within social norms interventions has the potential to achieve greater and more diffuse impact reaching others than those who were immediately and more intensively exposed to the intervention. If future community-based interventions intend to achieve social norms change within participants’ communities, they should equip participants with knowledge and skills to engage others in their network in transformative conversations. Intervention strategies that request participants to “adopt” other participants (as in the case of the CEP) can be of assistance here. The three interventions above offer three models of how this can be done, and a description of their programs exist in the literature (Cislaghi 2017; Clark et al. 2017; Denny and Hughes 2017a). While these interventions were on potentially controversial topics (such as FGC, IPV, or gender equality), we hypothesize that the organized diffusion strategy might be adapted and used for other outcomes, such as, for instance, parenting practices (Weber et al. 2017).

Our study has some limitations. The first is that the three case studies use different outcome measures to examine diffusion, the most direct being that of CEP. However, results from the three case studies converge supporting a robust finding of the presence of organized diffusion that is not confined to any one intervention type or outcome measure. The second limitation is related to the potential for participant bias. The diffusion effect might have only changed participants’ capacity to respond according to what they thought was expected of them. Data collection aimed to address this limitation by asking participants about their own practices and, in the case of the CEP case study, by triangulating information about injunctive family norms across family members. However, these limitations should be taken into account when interpreting study findings.

## Conclusion

Social norms can sustain harmful practices. Interventions being carried out in LMICs often integrate community-based dialogs to achieve social norms change but are criticized for not reaching change at scale. In this paper, we have analyzed data from three interventions, uncovering the potential of participant-led organized diffusion of knowledge and understandings across participants’ social network. Future research and practice shall increase our understanding of the most effective and ethical ways in which organized diffusion can help achieve greater social norms change to improve global health, well-being, and empowerment. Doing so has the potential to generate additional impact with little additional investment.
